# Exploration of the etiology of single small subcortical infarctions using high-resolution vessel wall MRI

**DOI:** 10.3389/fneur.2023.1179730

**Published:** 2023-05-30

**Authors:** Yutian Li, Quanzhi Feng, Congcong Wang, Xianchang Zhang, Liang Wan, Tong Han

**Affiliations:** ^1^Department of Radiology, Tianjin Huanhu Hospital, Tianjin University, Tianjin, China; ^2^Department of Radiology, Qingdao Women and Children's Hospital, Qingdao, Shandong, China; ^3^Department of Nuclear Medicine, The Affiliated Hospital of Qingdao University, Qingdao, Shandong, China; ^4^MR Collaboration, Siemens Healthineers Ltd., Beijing, China; ^5^Medical College, Tianjin University, Tianjin, China; ^6^Tianjin Key Laboratory of Cerebral Vascular and Neurodegenerative Diseases, Tianjin, China

**Keywords:** small subcortical infarction, high-resolution vessel wall imaging, stroke classification, lenticular artery, atherosclerotic plaque

## Abstract

**Objective:**

We aimed to explore imaging indicators for diagnosing the etiology of single small subcortical infarctions (SSI) using high-resolution vessel wall imaging (HR-VWI).

**Methods:**

Patients with acute isolated subcortical cerebral infarction were prospectively enrolled and classified as having large artery atherosclerosis (LAA), stroke of undetermined etiology (SUD), or small artery disease (SAD). The infarct information, the cerebral small vessel disease (CSVD) score, morphological characteristics of the lenticulostriate arteries (LSAs), and plaque characteristics were compared between the three groups.

**Results:**

Seventy seven patients were enrolled (30 LAA, 28 SUD, and 19 SAD). The total CSVD score of the LAA (*P* = 0.001) and SUD groups (*P* = 0.017) was significantly lower than that of the SAD group. The number and total length of LSA branches in the LAA and SUD groups were shorter than in the SAD group. Moreover, the total length laterality index (LI) of the LSAs in the LAA and SUD groups was greater than in the SAD group. The total CSVD score and LI of total length were independent predictors for the SUD and LAA groups. The remodeling index of the SUD group was significantly higher than that of the LAA group (*P* = 0.002); positive remodeling was dominant in the SUD group (60.7%), whereas remodeling in the LAA group was primarily non-positive (83.3%).

**Conclusions:**

SSI with and without plaques on the carrier artery may have different modes of pathogenesis. Patients with plaques may also have a coexisting mechanism of atherosclerosis.

## Introduction

A single subcortical infarction represents acute ischemic cerebral infarction in the blood supply area of the pons and basal ganglia (1, 2). The etiology of a single subcortical infarction remains controversial (3). Proposed causes include lipohyalinosis in lenticulostriate arteries (LSAs), termed small artery disease (SAD), or atherosclerosis in large parental arteries that obstruct a proximal branch or orifice of the LSAs, termed large artery atherosclerosis (LAA). The differentiation between the LAA and SAD mechanisms is clinically important because different etiologies lead to various consequences and necessitate distinct approaches for clinical management (4, 5).

However, it is difficult to distinguish the LAA from the SAD mechanism according to the clinical symptoms and signs. Moreover, traditional imaging techniques cannot visualize distal middle cerebral artery (MCA) plaques. Consequently, infarct size is the main discrimination index (6, 7). Small subcortical infarction (SSI) with a diameter <15 mm is classified by the SAD mechanism and large subcortical infarction (LSI) with a diameter >15 mm is classified by the LAA mechanism. However, this method does not consider the influence of atherosclerotic plaque in the affected carrier artery for SSI.

Research has recently shown that SSI without plaque in the affected carrier artery is caused by lipohyalinosis and fibrinoid degeneration (8). For SSI with plaque, the pathogenesis is controversial. TOAST (Trial of ORG 10172 in Acute Stroke Treatment) classified SSI with plaque as SAD because the carrier artery stenosis was <50% (9). The China Ischemic Stroke Subclassification (CISS) no longer considers the infarct lesion size and classifies all lesions with atherosclerotic plaques in carrier arteries as LAA (10).

The cerebral small vessel disease (CSVD) score is an effective neuroimaging method to evaluate the damage to the whole brain microstructure. Quantitative scoring by MRI imaging markers and grading is more conducive to accurately and comprehensively evaluating patients' overall vascular and brain injury status (11). Many studies have suggested that high-resolution vessel wall imaging (HR-VWI) can simultaneously image both the large vessel wall and the LSA lumen in one sequence, providing a repeatable quantitative index for plaque measurement and LSA quantification (12). Jiang et al. have used HR-VWI to evaluate the relationship between MCA plaques and the origin of LSAs. It was found that the distribution of plaques, rather than the nature of plaques, was the cause of the occurrence of single subcortical infarctions (13). The study of Ma et al. found that there was no significant difference in the display of LSAs between 3.0T HR-VWI and 7.0T MRA, although 7.0T TOF MRA could show longer segmentation of the few LSAs that were detected (14).

Therefore, the current study hypothesized that SSI with and without atherosclerotic plaques might have different modes of pathogenesis and carrier arterial plaque states, which could be reflected by HR-VWI. Furthermore, this study aimed to provide insight into SSI and discover effective imaging indicators for clinical diagnosis by comparing the related biological patient parameters of SSI and LSI, whose etiology was relatively clear.

## Materials and methods

### Patients

The Medical Ethics Committee of Tianjin Huanhu Hospital approved this study. All patients voluntarily gave informed consent for the study. Patients were retrospectively enrolled from January 2017 to February 2021. The inclusion criteria were: (1) diagnosed within 7 days of onset as having acute isolated subcortical cerebral infarction in the LSA's blood supply area (basal ganglia, corona radiata, and internal capsule), showing a high signal on diffusion-weighted imaging (DWI) and a low signal on apparent diffusion coefficient map; (2) the stenosis rate of the M1 segment of the MCA was <50% on CT angiography (CTA)/MR angiography (MRA). Patients were excluded if they: (1) had a history of stroke or transient ischemic attack within the previous 3 months; (2) showed ≥50% stenosis or occlusion of the MCA and internal carotid artery on CTA/MRA/digital subtraction angiography; (3) had bilateral subcortical infarcts or multiple infarcts on DWI; (4) had evidence of potential sources of cardiac embolism such as atrial fibrillation, and valvular heart disease; (5) had other causes of cerebral infarction, such as cerebral vasculitis, polycythemia, and thrombocythemia; (6) had a history of cerebral hemorrhage, brain tumor, or brain trauma; (7) received thrombolytic therapy after the onset; (8) had incomplete clinical data or poor MRI imaging that was difficult to evaluate.

**Figure 1:**
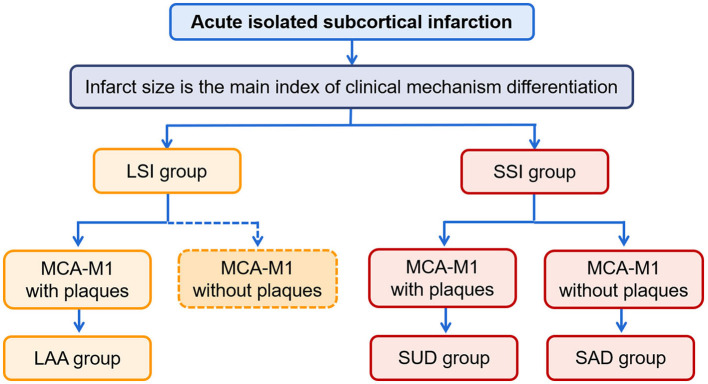
The procedure of classification. LSI, large subcortical infarction; SSI, small subcortical infarction; LAA, large artery atherosclerosis; SUD, stroke of undetermined etiology; SAD, small artery disease.

We divided the patients into LSI and SSI groups according to whether the diameter of the infarcts was >15 mm on DWI (6, 7). Furthermore, based on South Korea's modified TOAST classification (15), patients with atherosclerotic plaque detected by HR-VWI on the affected carrier artery (MCA-M1) in the LSI group were classified as having LAA; patients with plaques in the SSI group were classified as having SUD, and patients without plaques in the SSI group were classified as having SAD. The procedure for classification is shown in Figure 1. In addition, clinical information was collected from all patients, including demographic data, history associated with risk factors for stroke, and laboratory tests related to stroke risk factors.

### MRI protocols

All patients underwent routine MRI and HR-VWI on a Prisma or Skyra scanner (Siemens Healthcare, Erlangen, Germany) using identical sequences and parameters. The scan parameters are shown in Table 1. The CE^+^ HR-VWI scan was performed 5 min after injection of the contrast agent (0.1 mmol/kg, Magnevist, Bayer, Germany). Imaging data were evaluated and measured by multi-plane reconstruction using a Syngo Via workstation (Siemens Healthcare, Erlangen, Germany).

**Table 1:** MRI protocol in this study.

**Sequence**	**Acquisition orientation**	**FOV (mm^2^)**	**Slice thickness (mm)**	**Slices (*n*)**	**Spacing (mm)**	**TR/TE (ms)**	**Voxel size (mm^3^)**	**Acquisition time (s)**
DWI	Axial	240 × 240	5	21	1.5	5,200/80	0.9 × 0.9 × 5	38
T_2_WI	Axial	240 × 240	5	21	1.5	3,950/99	0.8 × 0.8 × 5	44
FLAIR	Axial	230 × 230	5	21	1.5	8,570/96	0.9 × 0.9 × 5	104
T_1_WI	Sagittal	270 × 270	5	13	1.5	240/2.47	0.8 × 0.8 × 5	35
T_1_WI	Axial	230 × 230	5	15	1.5	240/2.47	0.7 × 0.7 × 5	26
GRE	Axial	230 × 230	5	21	1.5	520/19.9	0.9 × 0.9 × 5	56
TOF-MRA	Axial	230 × 230	0.65	205	0	22/3.86	0.6 × 0.6 × 0.7	200
HR-VWI	Sagittal	230 × 230	0.55	240	0	900/15	0.6 × 0.6 × 0.6	446

FOV, field of view; TR, repetition time; TE, echo time; DWI, diffusion weighted imaging; T_2_WI, T_2_ weighed imaging; FLAIR, fluid attenuated inversion recovery; T_1_WI, T_1_ weighed imaging; GRE, gradient echo; TOF-MRA, time-of-flight magnetic resonance imaging angiography; HR-VWI, high-resolution vessel wall imaging.

### HR-VWI MRI information

Two experienced radiologists (HR and ME, who have worked in neuroradiology for 5 and 15 years, respectively) blinded to the clinical information independently evaluated the MRI images. If there were discrepancies between the two observers, a third senior physician (who has worked in neuroradiology for 20 years) participated in the evaluation.

### The CSVD score

The CSVD score from 0 to 4 included silent lacunar infarcts (SLIs), white matter hyperintensities (WMHs), enlarged perivascular spaces (EPVs), and cerebral microbleeds (CMBs). SLIs were defined as >3-but <15 mm in diameter in subcortical tissues, which was a cerebrospinal fluid signal on T2WI and no abnormal high signal on DWI (2, 11). If it was present, the total CSVD score was counted as 1 point. WMHs were defined as hyperintensities on T2WI surrounding the ventricles or in the deep white matter (2). According to the Fazekas scale, periventricular hyperintensity (PVH) was graded as 0 = absence, 1 = “caps” or “pencil-thin” lining, 2 = smooth “halo,” and 3 = irregular. Deep white-matter hyperintensity (DWMH) was graded as 0 = absence, 1 = punctate foci, 2 = partial fusion of punctate foci, and 3 = large confluent areas. In the presence of >2 points of DWMH or 3 points of PVM, the total CSVD score was counted as 1 point. EPVs were extensions of the extracerebral fluid space around the vessels (2). On all MRI sequences, the EPVs' signal intensity was similar to that of the cerebrospinal fluid. EPVs were graded as 0 = absence, 1 = 0–10; 2 = 10–20; 3 = 21–40; and 4 = ≥40 (16). In the presence of >2 points of EPVs, the total CSVD score was counted as 1 point. The CMBs were defined as >2 but <5 mm in diameter and hyperintense on gradient echo images in the cortical-subcortical junction, brainstem, basal ganglia, and cerebellum (2, 11). If it was present, the total CSVD score was counted as 1 point.

### Infarct measurement and location

**Figure 2:**
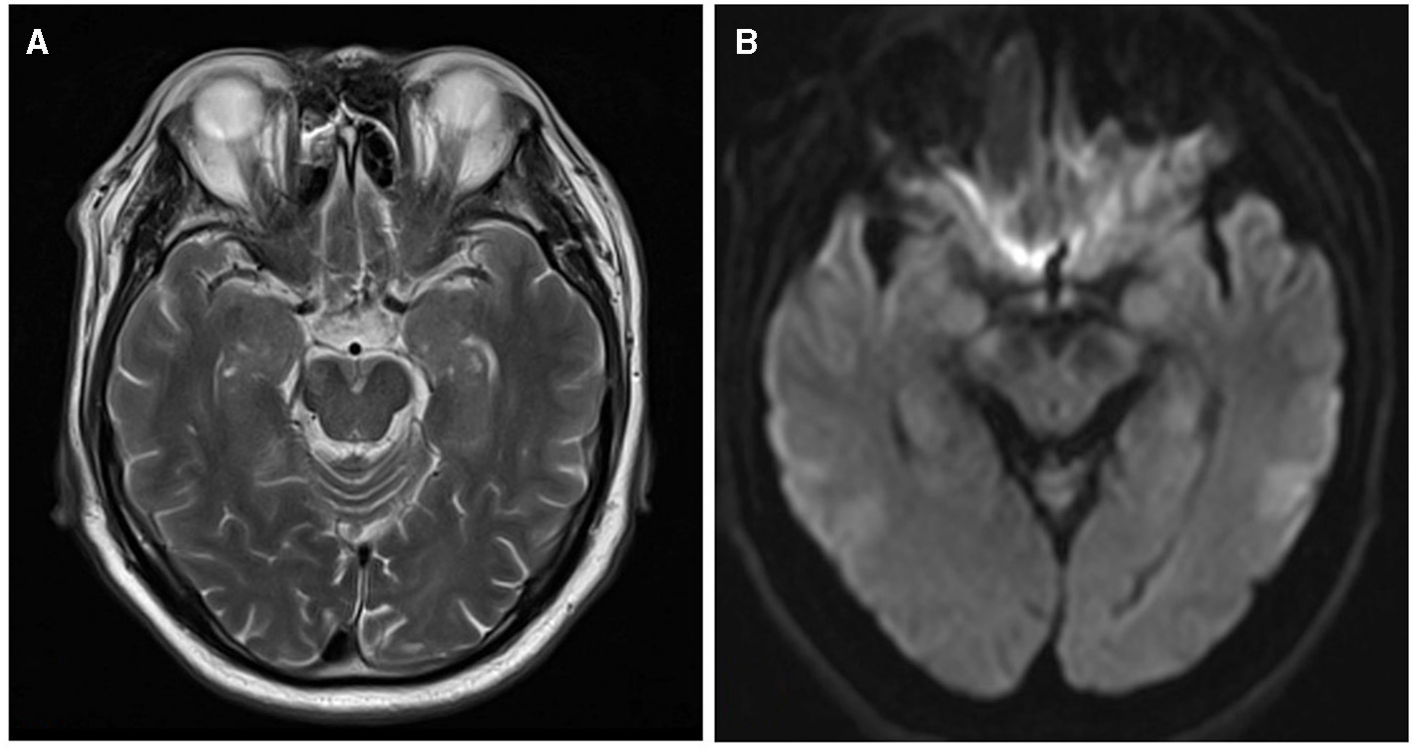
The layers on T2WI and DWI. **(A)** MCA-M1's normal flow void on T2WI. **(B)** The same layer on DWI is considered level 0.

On the axial DWI sequence, the largest slice of the lesion was selected, and the maximum diameter of the infarct was measured by two physicians separately (5, 17, 18). If there were discrepancies between two observers, they should work together to determine the final measurement. As shown in Figure 2, we found the MCA-M1's normal flow void on T2WI and considered the same layer on DWI as level 0. The next layer near the cephalic side was counted as the first level, and so on (19). Considering the infarction's medial lowest layer as the cutoff point, we defined the distal lesion as the lowest layer of the infarction lesion higher than the cutoff point, and others as the proximal lesions (17, 18, 20).

### LSAs morphometry

**Figure 3:**
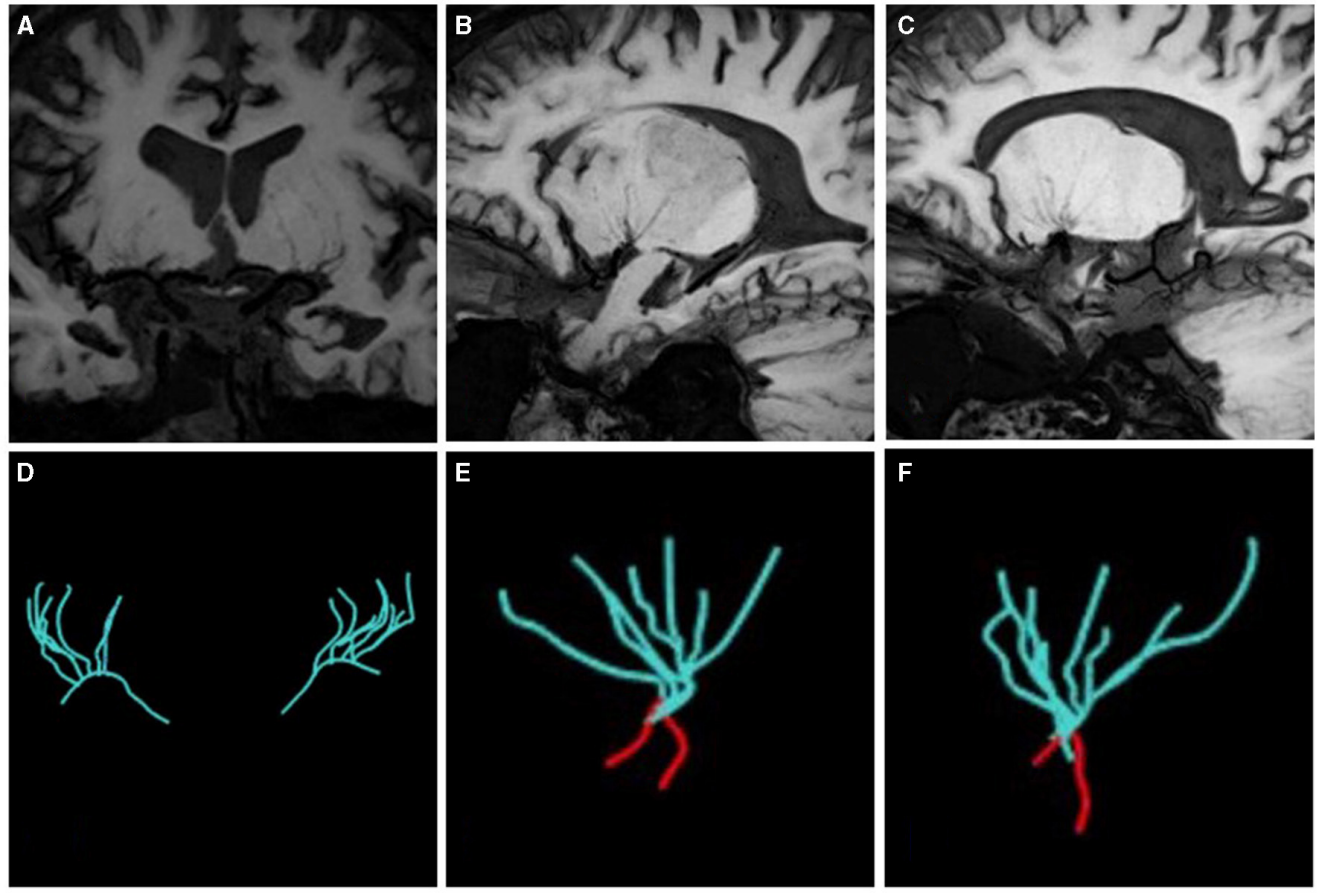
The vascular skeleton on HR-VWI (MinIP, 15 mm) and Sim Vascular. **(A, D)** The LSAs of two sides on the coronal view; **(B, E)** The LSAs of the left side on the sagittal view; **(C, F)** The LSAs of the right side on the sagittal view. The red curve represents the MCA.

As shown in Figure 3, the LSAs were traced using the software SimVascular (http://simvascular.github.io/) by referring to the method reported by Zhang et al. (21). Pathlines that could display the vascular skeleton were generated manually, thus reflecting the full length of the visual LSAs. When the vessel showed a continuous low signal and the contrast with the surrounding tissue was clear, tracking could continue. The part of the LSAs originating directly from MCA was defined as the stems. The portion of the LSA that originated from the LSA stems, or the LSAs without branches (single vessel), was defined as the branch. Trace LSA stems longer than 5 mm, but when the branches originating from MCA were shorter than 5 mm, each branch was counted separately because more than 75% of the branches originated from a common stem (22, 23). This study adopted the lateral index (LI). The LI was calculated as follows:

1. LI of stems = (number of stems _normal side_ – number of stems _lesion side_)/(number of stems _normal side_+ number of stems_lesion side_).
2. LI of branches = (number of branches_normal side_ – number of branches_lesion side_)/(number of branches_normal side_+ number of branches_lesion side_).
3. LI of total length = (total length_normal side_ –total length_lesion side_)/(total length_normal side_ + total length_lesion side_).

### Traditional parameters of the MCA-M1 plaque

**Figure 4:**
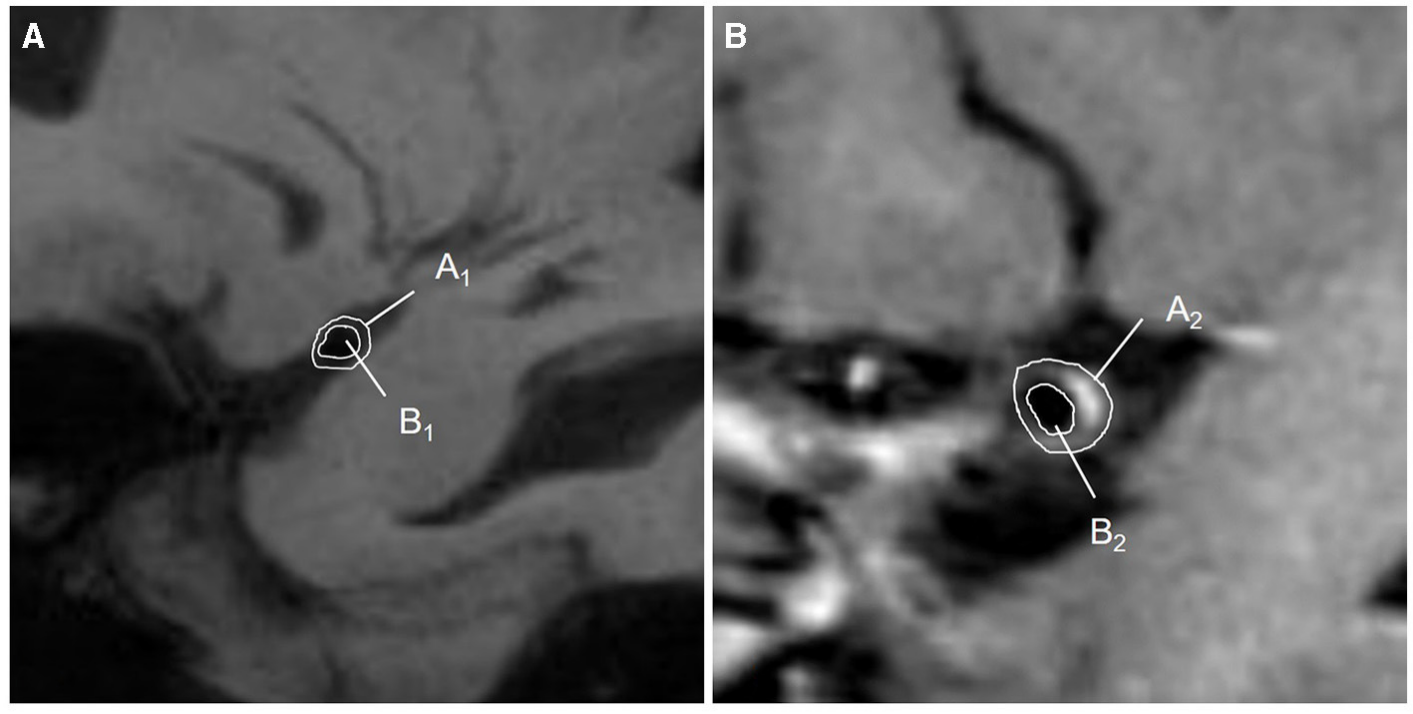
Diagram of quantitative measurements on HR-VWI. **(A)** The lumen at the reference sites was shown on HR-VWI. Area A_1_ = VA_1_; area B_1_ = LA_1_; WA_1_ = A_1_-B_1_; **(B)** The lumen at the lesion sites was shown on HR-VWI. Area A_2_ = VA_2_; area B_2_ = LA_2_; WA_2_ = A_2_-B_2_; plaque area = WA_2_ – WA_1_.VA, vessel area; LA, lumen area; WA, wall area.

A plaque was defined as eccentric or focal vessel wall thickening compared with its distal, proximal, or contralateral vessel segments (24). A culprit plaque was defined as the only plaque in the M1 segment of the affected side or the most stenotic one when there were multiple plaques in the vascular territory of the stroke (12, 25). If there were discrepancies in plaque recognition between two observers, they should invite a senior physician (who has worked in neuroradiology for 20 years) and work together to determine the final measurement. The plaque distribution on the short-axis view was segmented as the vessel wall's superior, dorsal, inferior, and ventral sides. Plaque enhancement was defined as a signal on enhanced plaque imaging higher than on an HR-VWI T1WI sequence and the contralateral vessel (5, 26). The thickest plaque was selected to measure the lumen diameter, and the lumen diameter at the reference sites was measured, too. Both the vessel area (VA) and lumen area (LA) at the lesion and reference sites were traced manually, as shown in Figure 4. Each indicator could be calculated as follows: (1) Wall area (WA) = VA-LA; (2) plaque area = WA _lesionsite_ – WA _referencesite_; (3) wall area index = WA _lesionsite_/WA _referencesite_; (4) plaque burden = (VA _lesionsite_ – LA _lesionsite_)/VA _lesionsite_ × 100% (27, 28); (5) stenosis rate = (1 – lumen diameter at the lesion site/lumen diameter at the reference site) × 100% (29). (6) The remodeling index (RI) was defined as the ratio between the LA at the lesion site and the reference site. If RI ≥1.05, it was defined as positive remodeling (PR); if RI ≤0.95, it was defined as negative remodeling (NR); if 0.95 < RI < 1.05, it was defined as intermediate remodeling. NR and intermediate remodeling were collectively called non-positive remodeling (non-PR) (30).

### Statistical analysis

All data were analyzed by commercial software (SPSS 26.0, IBM). For the quantitative data, normally distributed variables were summarized as means ± standard deviation (mean ± SD) and compared by a two-sample *t*-test or 1-way analysis of variance (ANOVA) test (The Bonferroni method was used for the *post hoc* test). The non-normally distributed data were expressed as the median and interquartile range and compared using the Mann–Whitey *U*-test or the Kruskal–Wallis *H*-test. Categorical variables were expressed as counts (percentages) and evaluated with the χ^2^ or Fisher exact test. Multivariate logistic regression analysis between the LAA and SUD groups was used to construct multivariate tests, which were adjusted for age, and gender and evaluated using odds ratios (OR) and 95% confidence intervals (CI). All analyses were two-sided, and *P* < 0.05 was considered statistically significant. ^*^P < 0.05, ^**^P < 0.01, and ^***^P < 0.001.

## Results

**Figure 5:**
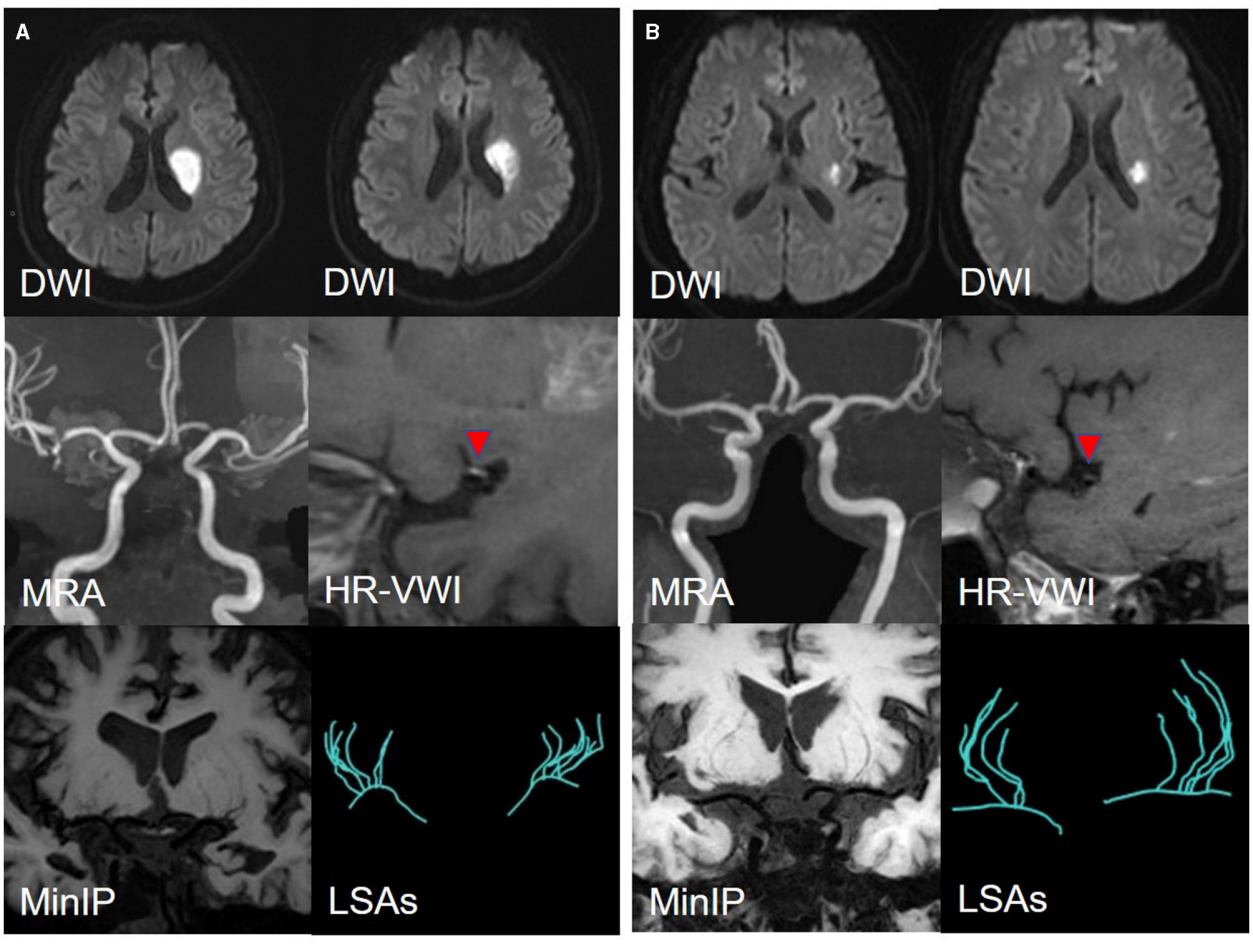
**(A)** A representative case of the LAA group: a 35-year-male. DWI shows an isolated subcortical cerebral infarction located in the LSAs territory, and the maximum axial diameter is 24 mm. MRA shows no obvious stenosis of the MCA-M1 on either side. HR-VWI shows the plaque located on the superior side of the MCA-M1. MinIP and the Sim Vascular show the LSAs' vascular skeleton. **(B)** A representative case of the SUD group: a 55-year-male. DWI shows an isolated subcortical cerebral infarction located in the LSAs territory and the maximum axial diameter is 12 mm. MRA shows no obvious stenosis of the MCA on either side. HR-VWI shows the plaque located on the superior side of the MCA-M1. MinIP and the Sim Vascular show the LSAs' vascular skeleton.

**Figure 6:**
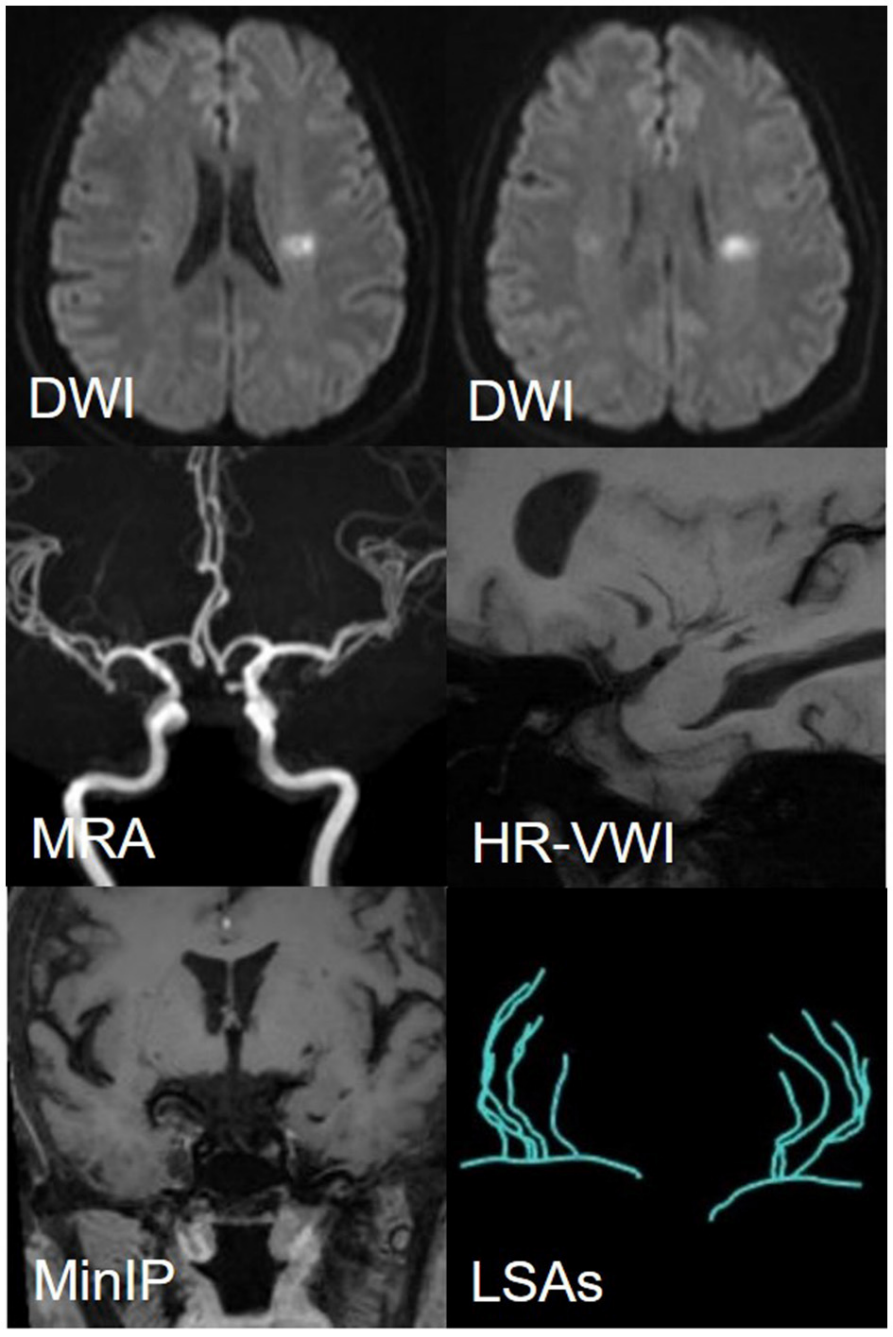
A representative case of the SAD group: a 56-year-female. DWI shows an isolated subcortical cerebral infarction located in the LSAs territory, and the maximum axial diameter is 11 mm. MRA shows no obvious stenosis of the MCA on either side. HR-VWI shows no plaques located on the MCA-M1. MinIP and the Sim Vascular show the LSAs' vascular skeleton.

Among 110 patients, 18 were excluded, including nine due to cardiogenic embolism, one due to arteritis, four due to MCA-M1 stenosis rate >50%, and four due to poor image quality. Ninety-two patients remained. Of these, 45 were allocated to the LSI group and 47 to the SSI group. In the LSI group, 30 patients were allocated to the LAA group. In the SSI group, 28 were allocated to the SUD group and 19 to the SAD group. Ultimately, 77 patients were enrolled, and three representative cases from each group are shown in Figures 5, 6. As shown in Table 2, there were no statistical differences in risk factors or laboratory tests between the three groups (*P* > 0.05).

**Table 2:** Risk factors and laboratory test comparison between the three groups.

	**LAA (*n* = 30)**	**SUD (*n* = 28)**	**SAD (*n* = 19)**	**χ^2^ or *Z***	***P-*value**
**Risk factors**					
Age (year)	56.5 (39, 63)	54 (40.25, 63)	53 (37, 61)	0.256	0.88
Male (*n*, %)	19 (63.3)	22 (78.6)	16 (84.2)	2.892	0.243
Smoking (*n*, %)	10 (33)	14 (50)	7 (36.8)	1.795	0.408
Hypertension (*n*, %)	22 (73.3)	21 (75)	13 (68.4)	0.256	0.88
Diabetes mellitus (*n*, %)	8 (26.7)	6 (21.4)	3 (15.8)	0.778	0.690
Hyperlipidemia (*n*, %)	12 (40.0)	13 (46.4)	10 (52.6)	0.765	0.682
**Laboratory tests**					
SBP (mmHg)	153 (138.0, 170.5)	149 (137.8, 161.5)	153 (137.0, 160.0)	0.320	0.852
DBP (mmHg)	89.57 ± 3.258	94.46 ± 2.428	94.47 ± 2.995	0.971	0.383
TG (mmol/L)	1.73 (1.43, 2.52)	1.64 (1.18, 2.00)	1.28 (0.97, 1.97)	3.402	0.182
TC (mmol/L)	4.54 (3.90, 5.15)	4.77 (3.99, 5.26)	4.73 (3.49, 5.10)	1.085	0.581
HDL (mmol/L)	0.99 (0.91, 1.13)	1.00 (0.89, 1.08)	1.11 (0.86, 1.27)	0.893	0.640
LDL (mmol/L)	3.02 (2.56, 3.58)	3.19 (2.6, 3.55)	2.81 (2.03, 3.33)	2.343	0.310
FBG (mmol/L)	5.65 (4.66, 6.52)	5.22 (4.61, 6.24)	5.00 (4.28, 6.23)	0.908	0.635

LAA, large artery atherosclerosis; SUD, stroke of undetermined etiology; SAD, small artery disease; SBP, systolic blood pressure; DBP, diastolic blood pressure; TG, triglyceride; TC, total cholesterol; HDL, high density lipoprotein; LDL, low density lipoprotein; FBG, fasting blood-glucose.

The total CSVD score was smaller in the LAA and SUD groups than in the SAD group (*Z* = −22.472, *P* = 0.001; *Z* = −17.462, *P* = 0.017; Table 3). Moreover, the incidence of EPVs was lower in the LAA and SUD groups than in the SAD group (χ^2^ = 11.495, *P* = 0.001; χ^2^ = 16.505, *P* = 0.001).

**Table 3:** Total CSVD score and infarction information comparison between the three groups.

	**LAA (*n* = 30)**	**SUD (*n* = 28)**	**SAD (*n* = 19)**	**χ^2^ *or Z***	***P*** **-value**			
						**LAA vs. SUD**	**LAA vs. SAD**	**SUD vs. SAD**
**Total CSVD score**								
Total CSVD score	1 (0, 1.25)	1 (0.25, 2)	2 (1, 3)	13.518	0.001	0.370	0.001	0.017
SLIs (*n*, %)	17 (56.7)	18 (64.3)	12 (63.2)	0.401	0.818			
WMHs (*n*, %)	5 (16.7)	2 (7.1)	9 (47.4)	10.435	0.004	0.425	0.027	0.445
EPVs (*n*, %)	6 (20.0)	8 (28.6)	15 (78.9)	18.766	0.001	0.446	0.001	0.001
CMBs (*n*, %)	4 (13.3)	6 (21.4)	2 (10.5)	1.118	0.571			
**Infarction**								
Total layers (*n*)	5 (4, 6)	2 (2, 4)	2 (1, 3)	34.254	0.001	0.001	0.001	0.463
Lowest layer (*n*)	2 (1, 3)	3 (1,4)	3 (3, 4)	10.355	0.006	0.359	0.004	0.219
Proximal lesions (*n*, %)	21 (70)	13 (46.4)	4 (21.1)	11.300	0.004	0.069	0.001	0.076

LAA, large artery atherosclerosis; SUD, stroke of undetermined etiology; SAD, small artery disease; CSVD, total cerebral small vessel disease; SLIs, silent lacunar infarcts; WMHs, white matter hyperintensities; EPVs, enlarged perivascular spaces; CMBs, cerebral microbleeds.

Of all the patients, the median lowest layer of lesions was at the 2.5th level. Patients with the lowest layer of the lesions <2.5th were defined as having the proximal lesion and others as having the distal lesion. The lowest layer of lesions was lower in the LAA group than in the SAD group (*Z* = −20.511, *P* = 0.004). The incidence of the proximal lesions was higher in the LAA than in the SAD group (Table 3).

As shown in Table 4, there were no statistically significant differences in the number of stems (*Z* = 5.661, *P* = 0.059) among the three groups or the number of LSA branches (*Z* = 1.745, *P* = 0.388) on the normal side, whereas the LAA group had a shorter total length than the SAD group (*P* = 0.007) on the normal side.

**Table 4:** LSAs morphological comparison between three groups.

	**LAA (*n* = 30)**	**SUD (*n* = 28)**	**SAD (*n* = 19)**	***P*** **-value**			
					**LAA vs. SUD**	**LAA vs. SAD**	**SUD vs. SAD**
**Normal side**							
LSAs stems (*n*)	4 (4, 5)	5 (4, 5)	5 (4, 5)	0.059			
LSA branches (*n*)	6 (5.75, 7)	6 (5, 7)	7 (6, 8)	0.388			
Total length of LSAs (mm)	124.61 ± 6.33	132.63 ± 6.02	152.19 ± 8.18	0.025	0.371	0.007	0.056
**Symptomatic side**							
LSAs stems (*n*)	4 (3, 5)	4 (3, 4)	5 (4, 6)	0.017	0.819	0.175	0.014
LSA branches (*n*)	6 (5, 6)	5 (4.25, 6)	7 (6, 8)	0.006	0.402	0.049	0.006
Total length of LSAs (mm)	89.85 (85.2, 115.23)	101.05 (87.4, 128.03)	139.9 (120.2, 172.4)	0.001	0.865	0.000	0.002
LI of stems	0 (0, 0.142)	0.11 (0, 0.143)	0 (0, 0.11)	0.120			
LI of branches	0.071 (0, 0.091)	0.954 (0, 0.143)	0 (0, 0.067)	0.002	0.317	0.088	0.001
LI of total length	0.01 ± 0.017	0.10 ± 0.014	0.02 ± 0.07	0.001	0.813	0.000	0.000

LAA, large artery atherosclerosis; SUD, stroke of undetermined etiology; SAD, small artery disease; LSAs, lenticulostriate arteries; LI, laterality index.

**Figure 7:**
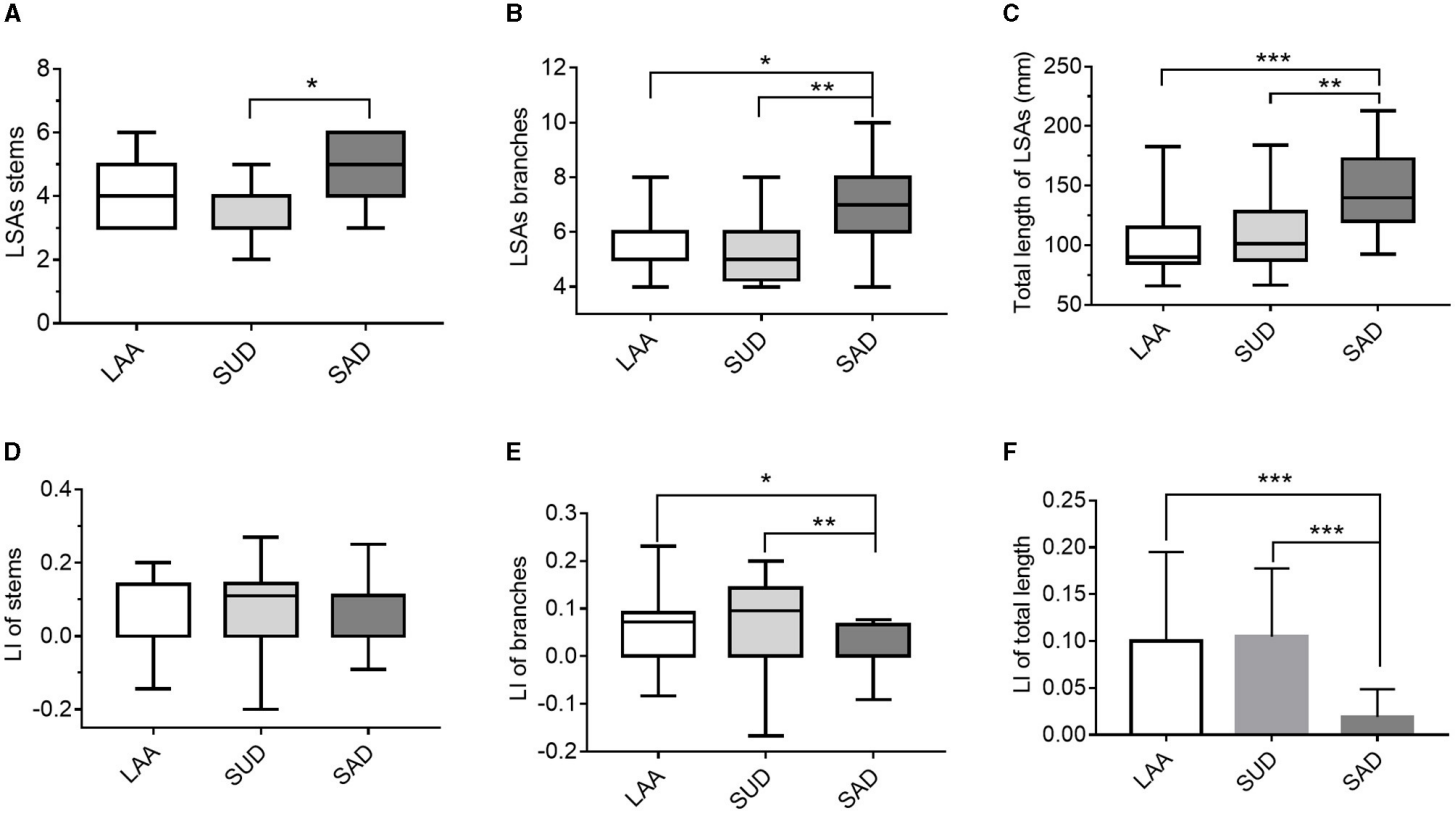
Comparison of the LSAs' morphological changes between the LAA, SUD, and SAD groups according to **(A)** LSAs stems; **(B)** LSA branches; **(C)** total length of the LSAs; **(D)** LI of stems; **(E)** LI of branches; **(F)** LI of length. LAA, large artery atherosclerosis; SUD, stroke of undetermined etiology; SAD, small artery disease. LSAs, lenticulostriate arteries; LI, laterality index. *P < 0.05, **P < 0.01, and ***P < 0.001.

As shown in Table 4 and Figure 7, the number of stems was smaller in the SUD group than in the SAD group. The SAD group had a larger number of LSA branches than the SUD (*Z* = −20.085, *P* = 0.006) and LAA groups (*Z* = −15.296, *P* = 0.049). There was a significant reduction in the LI of branches of the LSAs in the SAD group compared with the SUD and LAA groups (*Z* =23.34, *P* = 0.001; *Z* =14.014, *P* = 0.088). The SUD and LAA groups had a shorter total length than the SAD group (*Z* = −22.449, *P* = 0.002; *Z* = −28.692, *P* = 0.000). The LI of the total length of the LSAs in the SUD and LAA groups was greater than in the SAD group (*P* = 0.000; *P* = 0.000).

The factors with statistical significance in the univariate analysis, including gender and age, were analyzed by multivariate logistic regression analysis, as shown in Table 5. The result showed that taking the SAD group as a control, the total CSVD score had a statistically significant effect on the LAA and SUD groups (OR: 0.200, 95%CI: 0.054–0.743, *P* = 0.016; OR: 0.226, 95%CI: 0.062–0.826, *P* = 0.025). LI of total length had a statistically significant effect on the LAA and SUD groups (OR: >999.999, 95% CI: 473.933 to >999.999, *P* = 0.017; OR: >999.999, 95% CI: 46.305 to >999.999, *P* = 0.025).

**Table 5:** Multivariate logistic regression analysis between the LAA and SUD groups.

	**LAA (*****n*** = **30)**		**SUD (*****n*** = **28)**	
	**OR (95% CI)**	***P*** **-value**	**OR (95% CI)**	***P*** **-value**
Age	0.931 (0.848 to 1.021)	0.128	0.976 (0.895 to 1.063)	0.572
Gender	1.270 (0.435 to 3.708)	0.662	0.151 (0.008 to 2.935)	0.212
Total CSVD score	0.200 (0.054 to 0.743)	0.016	0.226 (0.062 to 0.826)	0.025
Lowest layer	0.582 (0.103 to 3.292)	0.540	0.437 (0.080 to 2.392)	0.340
Proximal lesions	0.209 (0.004 to 10.778)	0.436	1.406 (0.029 to 68.651)	0.864
LSA branches	1.248 (0.399 to 3.906)	0.703	0.644 (0.210 to 1.974)	0.441
Total length of LSAs	0.983 (0.943 to 1.024)	0.401	1.003 (0.962 to 1.045)	0.895
LI of branches	0.001 (<0.001 to >999.999)	0.516	0.422 (<0.001 to >999.999)	0.920
LI of total length	>999.999 (473.933 to >999.999)	0.017	>999.999 (46.305 to >999.999)	0.025

The SAD group was taken as control group. 95% CI, 95% confidence interval; OR, odds ratio; LAA, large artery atherosclerosis; SUD, stroke of undetermined etiology; CSVD, total cerebral small vessel disease; LSAs, lenticulostriate arteries; LI, laterality index.

As shown in Table 6, the plaque distribution was approximately the same between the LAA and SUD groups (χ^2^ = 0.031, *P* = 0.86), and those plaques were more frequently located on the superior and dorsal sides of the vessel wall (LAA, 70%; SUD, 67.9%). The wall area index was higher in the SUD group than in the LAA group (*Z* = −2.085, *P* = 0.037). A smaller plaque burden (*t* = 2.289, *P* = 0.026) was shown in the SUD group. The SUD group demonstrated smaller plaque enhancement (39.3 vs. 66.7%, χ^2^ = 4.364, *P* = 0.037). The stenosis rate was higher in the SUD than in the LAA group (*Z* = −2.940, *P* = 0.003). As shown in Table 7, the SUD group had a higher remodeling index than the LAA group (*Z* = −3.174, *P* = 0.002). PR (60.7%) was more frequently observed in the SUD group, whereas non-PR (83.3%) was more frequently observed in the LAA group.

**Table 6:** MCA plaque characteristics comparison between LAA and SUD groups.

	**LAA (*n* = 30)**	**SUD (*n* = 28)**	***Z* or *t***	***P*-value**
**Wall morphology**				
Vessel area (mm^2^)	16.19 ± 0.61	16.98 ± 0.68	−0.868	0.389
Lumen area (mm^2^)	4.53 ± 0.25	5.20 ± 0.21	−2.001	0.050
Wall area (mm^2^)	11.41 (10.41, 12.91)	11.57 (9.71, 12.84)	−0.342	0.732
Wall area index	1.09 (1.04, 1.16)	1.15 (1.10, 1.19)	−2.085	0.037
**Plaque characteristics**				
Plaque area (mm^2^)	0.940 (0.435, 1.820)	1.395 (1.185, 1.740)	−1.642	0.101
Plaque burden (%)	72.3 ± 1.0	68.9 ± 1.1	2.289	0.026
Number of quadrants (*n*)	2 (1, 3.5)	2 (1, 2.75)	−0.338	0.736
Plaques on superior and dorsal sides (*n*, %)	21 (70)	19 (67.9)	0.031	0.860
Enhancement (*n*, %)	20 (66.7)	11 (39.3)	4.364	0.037
Stenosis rate (%)	18.2 (12.6, 27.4)	10.5 (6.7, 17.7)	−2.940	0.003
Remodeling index	0.972 (0.947, 1.035)	1.057 (1.000, 1.070)	−3.174	0.002

LAA, large artery atherosclerosis; SUD, stroke of undetermined etiology.

**Table 7:** Artery remodeling modes of atherosclerosis for LAA and SUD group.

	**LAA (*n* = 30)**	**SUD (*n* = 28)**	**χ^2^ or *Z***	***P*-value**
Remodeling index	0.972 (0.947, 1.035)	1.057 (1.000, 1.070)	−3.174	0.002
Remodeling mode			12.444	0.002
Positive remodeling (*n*, %)	5 (16.7)	17 (60.7)		
Intermediate remodeling (*n*, %)	17 (56.7)	9 (32.1)		
Negative remodeling (*n*, %)	8 (26.7)	2 (7.1)		

LAA, large artery atherosclerosis; SUD, stroke of undetermined etiology.

## Discussion

In this study, we found that proximal lesions often occurred in LSI patients with plaques compared to SSI patients without plaques, reflecting the different etiology. Moreover, a significant reduction in the total CSVD score, the total length of LSAs, and a significant rise in the LI of LSAs' total length were observed in SSI with plaques. Moreover, the lower total CSVD score and the larger LI of total length have heightened the risk of SSI with plaques, similar to the LSI patients with plaques. In addition, most LSI patients with plaques showed non-positive remodeling, whereas most SSI patients with plaques showed positive remodeling. These results suggested differences in the etiologies between SSI patients with and without plaques. Plaque characteristics may play an essential role in the etiology of SSI with plaques.

The total CSVD score reflected long-term small vessel damage in the whole brain and a marked increase in the brain's susceptibility to ischemia, hypoxia, and other injuries (11). The higher the score, the more severe the damage to small vessels. Conventional SAD is referred to as lacunar infarction. According to the TOAST classification, SAD was defined as a lesion with an infarction size of <15 mm and no stenosis in the carrier artery. Patients usually have a history of hypertension and diabetes mellitus. Therefore, the patient's condition is mainly caused by lipohyalinosis and fibrinoid for LSAs. This study showed no significant difference between the LAA and SUD groups, though the total CSVD score was smaller in the LAA and SUD groups than in the SAD group. Multivariate logistic regression analysis showed that a smaller CVSD score increased the risk of the SUD group, which was consistent with changes in the LAA group. This finding demonstrated that the degree of small vessel damage in the SUD patients was less than in the SAD patients but the same as in the LAA patients, suggesting that the location of the blood vessels involved in the lesion differed from that in the SAD patients.

In terms of infarction, the incidence of proximal lesions was less in the LAA (70%) groups than in the SAD group (21.1%). However, the LAA group had a higher incidence of proximal lesions, consistent with branch atheromatous disease (BAD) characteristics. There were three mechanisms for perforating artery stenosis or occlusion by atherosclerotic lesions (3): (I) the atherosclerotic lesions in MCA-M1 occluded the origin of a perforating artery; (II) the atherosclerotic lesions in MCA-M1 extended to a perforating artery origin; (III) the atherosclerotic lesions located in a perforating artery origin led to its narrowing or occlusion. Therefore, the atherosclerotic lesions in MCA-M1 affected a perforating artery's origin or proximal aspect, resulting in a lower layer of the lesions, most commonly defined as “the proximal lesion.” Yang et al. (17) divided the lacunar infarction according to the cutoff considered by the median of the lowest layer of the infarction lesions and the mean of the lesion size, finding the BAD group had larger infarct lesions and a lower level of the lowest layer of the infarction lesions, consistent with the current study. The incidence of proximal lesions in the SUD group was not only slightly higher than that of the LAA group but also slightly lower than that of the SAD group, but there was no statistically significant difference. This would indicate that the incidence of proximal lesions in the SSI should not be used to distinguish its mechanism.

This study used HR-VWI to explore LSAs characteristics. However, the voxel of HR-VWI was 0.6 mm × 0.6 mm × 0.6 mm, and the diameters of the LSAs were 0.3–0.8 mm (31). Therefore, HR-VWI could only display part of the LSAs' shapes but not changes in the vascular walls. Fortunately, some studies proved that the visualization of LSAs in HR-VWI was consistent with that in MRA at 7T (21). However, in addition to DSA and 7T-TOF MRA, most of the studies projected the three-dimensional distributed LSAs on a two-dimensional minimum intensity projection with 10–15 mm thickness before measuring the number and length of the LSAs. Therefore, this method cannot clearly show the relationship between the various trunks and branches of LSAs and can only display the projection length of LSAs in the coronal position instead of visualizing the actual shape of LSAs in three-dimensional space. To overcome this limitation, we followed a previous study using the mature software Sim Vascular to construct the vascular skeleton of LSAs in multiple directions, truly reflecting the three-dimensional spatial distribution of LSAs (21). This method could help more accurately depict the direction and shape of the perforating arteries, thus obtaining a more accurate quantification of the LSA's morphology.

Jiang et al. (13) studied 40 single subcortical infarction patients without relevant MCA disease and found that the average length and distance of the LSAs on the symptomatic side were shorter than on the normal side. However, there was no systematic comparison of the LSAs in patients with different types of infarctions. Using atherosclerotic lesions with relatively clear etiology as a reference, the current study systematically analyzed the LSAs in patients with different types of SSI, particularly patients with SSI with plaques. In the LAA group, the atherosclerotic lesions in the carrier artery occluded the origin of a perforating artery or extended to a perforating artery origin, involving a higher degree of LSAs. So, the LAA group exhibited shorter numbers of LSA branches and a shorter LSA length, suggesting that LSAs were affected and the total length of the visualized LSAs was short. In addition, SAD tended toward lipohyalinosis, and fibrinoid and vasculopathy occurred in the distal segment of LSAs. Compared with the LAA group, the total length of the lenticulostriate artery was less affected. In this study, the number of LSA branches and LSA length were longer in the SAD group compared with the LAA group, reflecting that LSAs were less affected and the diameters of the vessels involved were smaller, suggesting the SAD group tended toward lipid hyalinization. These findings were consistent with previous studies (12, 23). Notably, the number of LSA branches and total visualized LSA length in the SUD group differed from those in the SAD group because, although the infarcts were the same size in both groups, the degree of LSA involvement was not identical. Moreover, there were no statistically significant differences in the number of LSA branches and total length between the SUD and LAA groups, suggesting that the LSAs involved were similar and the pathogenesis was closer.

LI was commonly used in functional magnetic resonance imaging, indicating an asymmetry of activation ranging from −1 to 1 (32). LI was introduced in this study to reflect the asymmetry of LSA's involvement, with a difference between the normal and symptomatic sides. The LAA group exhibited a more substantial LI of branches of the LSAs and a larger total LI of lengths of the LSAs compared with the SAD group. This finding reflected the different modes of pathogenesis in the two groups. Specifically, the LAA group tended to the atherosclerotic mechanism, and the SAD group tended to the small artery disease mechanism. More importantly, there was a statistically significant difference in LI between the SUD and SAD groups but not between the SUD and LAA groups, suggesting that LSAs' involvement in the SUD and LAA groups was analogous. In addition, although there were atherosclerotic plaques in the carrier arteries of the SUD and LAA groups, the infarct sizes were different in the two groups, suggesting that atherosclerotic plaques may play different roles in the pathogenesis of the two groups. Multiple logistic regression analysis showed that using the SAD group as a control group, LI of total length was an independent predictor for SUD and LAA groups, and a larger LI of total length increased the risk of SUD and LAA groups. Not only did this suggest some similarity between the SUD and LAA groups, but it also suggested that the LI of total length displayed by HR-VWI may become an imaging marker for identifying the mechanism of single subcortical infarctions.

The remodeling index was significantly different between the SUD and LAA groups. The LAA group demonstrated primarily non-positive remodeling, whereas the SUD group showed mostly positive remodeling. This finding may have been the primary reason for the inconsistency in infarct size between the two groups. Arterial remodeling was initially used to describe coronary stenosis (“Glagov” phenomenon) (33) and reflected compensatory changes made by arteries in response to various stimuli. Previous studies showed that PR was correlated with unstable plaques and was one of the risk factors for stroke. Xu et al. (34) observed patients with symptomatic and asymptomatic MCA atherosclerotic stenosis and found that the symptomatic group had a greater remodeling ratio and was characterized primarily by PR. Liao et al. (5) enrolled 50 patients with acute perforating artery infarcts in the anterior circulation, finding a similar characterization between BAD and lacunar infarct patients. However, BAD patients primarily had relatively stable plaques, whereas lacunar infarct patients had unstable plaques. This result was similar to the current study, suggesting that microemboli produced by unstable plaques occluded the perforating arteries in the SUD group. This finding also explained how the SUD group started suddenly, with milder clinical symptoms and more common recurrences.

Plaque burden includes not only the degree of vascular stenosis but also wall areas and plaque areas. Shi et al. (27) showed that the progression of plaque burden was an independent predictor of stroke recurrence. Fakih et al. (24) showed a positive correlation between plaque burden and the degree of stenosis shown by HR-VWI. It was found that patients in the LAA group had a relatively large plaque burden. It should be noted that the LAA group has a higher degree of stenosis compared to the SUD group. However, this study only observed patients with first acute ischemic stroke. It is necessary to conduct longitudinal studies using HR-VWI on the association between plaque burden and stroke recurrence.

There were several limitations in this study. First, this was a single-center study; most patients were northern Chinese people. Whether the results apply to other populations needs further confirmation. Second, this study was cross-sectional; therefore, future longitudinal observation of patient outcomes and recurrence is needed. Finally, this study only analyzed the morphological characteristics of plaques, but the histological characteristics of plaques, such as intraplaque hemorrhage and fibrous cap structure, were not assessed.

Therefore, atherosclerotic plaques in SSI patients may develop with a coexisting atherosclerotic mechanism and demonstrate a lower total CVSD score and shorter total LSA length. This finding is consistent with the LSI patients. However, the plaques in LSI and SSI were different: those in LSI showed primarily non-positive remodeling, whereas those in SSI demonstrated mostly positive remodeling. The microemboli produced by the positively reconstructed unstable plaques may be the pathological mechanism underlying the appearance of small infarcts. Finally, HR-VWI can simultaneously depict the plaque on the carrier and perforator arteries, which may provide reproducible quantitative indicators for distinguishing SSI from different modes of pathogenesis.

## Data availability statement

The original contributions presented in the study are included in the article/supplementary material, further inquiries can be directed to the corresponding authors.

## Ethics statement

The studies involving human participants were reviewed and approved by the Medical Ethics Committee of Tianjin Huanhu Hospital. The patients/participants provided their written informed consent to participate in this study. Written informed consent was obtained from the individual(s) for the publication of any potentially identifiable images or data included in this article.

## Author contributions

Material preparation and data collection were performed by YL, QF, CW, and XZ. Data analysis was performed by YL, CW, and TH. The first draft of the manuscript was written by YL. All authors contributed to the study conception and design, commented on previous versions of the manuscript, read, and approved the final manuscript.
